# Mechanistic Study of Substituent Effect on Photoinduced O-C Bond Activation in Polycarbonate

**DOI:** 10.3390/molecules30081839

**Published:** 2025-04-19

**Authors:** Xiao Huang, Yuuichi Orimoto, Yuriko Aoki

**Affiliations:** 1 Department of Interdisciplinary Engineering Sciences, Chemistry and Materials Science, Interdisciplinary Graduate School of Engineering Sciences, Kyushu University, 6-1 Kasuga Park, Fukuoka 816-8580, Japan; huang.xiao.932@s.kyushu-u.ac.jp; 2 Department of Material Sciences, Faculty of Engineering Sciences, Kyushu University, 6-1 Kasuga Park, Fukuoka 816-8580, Japan; orimoto.yuuichi.888@m.kyushu-u.ac.jp

**Keywords:** polycarbonate, bond cleavage, DFT/TDDFT

## Abstract

Photodegradation of polycarbonate (PC) is investigated based on quantum chemical methods with PC models to clarify the effect of substituents at different positions of phenyl rings on the carbonate O-C bond cleavage. Compared to the results without substituents on phenyl rings, the breakage of the carbonate O-C bond is promoted or suppressed when the electron-donating or electron-withdrawing group is placed on the *meta*- or *ortho*-positions of the *gem*-dimethyl groups of phenyl rings, respectively. Moreover, the promotion and suppression of carbonate O-C bond scission are more significant if the substituents are located on the *ortho*-positions of the *gem*-dimethyl groups.

## 1. Introduction

Polycarbonate (PC) is known for its excellent physical properties, including optical clarity, high toughness, high impact resistance, and tensile strength [1,2]. As a result, it has gained widespread applications, such as in the medical industry, construction and engineering, electronics, and so on. Unfortunately, these utilizations and the stability of PC products could be restricted due to the decrease in its desirable performances caused by excessive exposure to sunlight or ultraviolet (UV) light [1,2]. Furthermore, when PC plastic products lose their designated functions, this may lead to pollution if not properly disposed of [3]. To boost the durability of PC products or facilitate the degradation of discarded PC plastics, it is crucial to examine the influence of light on PC photodegradation, which has been widely explored through experimental studies for years [4,5,6,7,8,9]. Structural modifications, such as the replacement of phenyl hydrogen atoms with electron-donating or electron-withdrawing groups, are typically used methods to change PC performance experimentally [10,11,12].

At present, only a limited number of theoretical studies have concentrated on aromatic esters [13,14,15] and PC photodegradation [16,17], especially those focusing on the influence of substituents of phenyl rings on carbonate damage. Recently, employing quantum chemical methods, our group investigated the mechanism of carbonate O-C bond cleavage using PC models, including the bisphenol-A hydrogen carbonate (BPAHC) model [16] and substituted BPAHC models with -NH_2_ and -NO_2_ groups at *meta*-positions of the *gem*-dimethyl groups of phenyl rings [17]. According to the results of BPAHC [16], there are two factors affecting carbonate O-C bond cleavage. First, the electronic excitation from oxygen lone pairs to the phenyl (near the carbonate group) π* orbital can lead to the formation of a quinoid-like structure via the enchantment of C-C in-phase overlaps. Second, the electronic excitation from oxygen lone pairs to the carbonate π* orbital can cause the extension of the carbonate O-C bond through the strengthened O-C out-of-phase overlap. The cooperative effect of both the above excitations based on the geometry of the ground state (GS) finally leads to the cleavage of the carbonate O-C bond, as observed in the geometry of the excited state (ES). Compared to the results of BPAHC, the analysis of the -NH_2_ substituted BPAHC model suggests that the cleavage of the carbonate O-C bond can be promoted under the influence of the electron-donating -NH_2_ group at *meta*-positions of the *gem*-dimethyl groups of the phenyl rings, because the O-C bond elongation has a greater driving force (relative to that of BPAHC) caused by the reinforced excitation from oxygen lone pairs to the carbonate π* orbital [17]. On the contrary, the suppression of the carbonate O-C bond scission is affected by the electron-withdrawing -NO_2_ group at *meta*-positions of the *gem*-dimethyl groups of the phenyl rings due to the almost diminished excitation from oxygen lone pairs to the carbonate π* orbital [17].

To further shed more light on the substituent effect on the carbonate O-C bond cleavage of PC, other PC models were investigated, where the -NH_2_ and -NO_2_ groups acted as the substituents at *ortho*-positions of the *gem*-dimethyl groups of the phenyl ring. The present work aims to clarify how the electron-donating and -withdrawing groups at different positions of phenyl rings affect the carbonate O-C bond cleavage. Ultimately, it is possible to screen out light-resistant PC material or degradable PC material, obtaining future directions for PC chemical structure modification based on the theoretical results.

## 2. Results and Discussion

**Figure 1:**
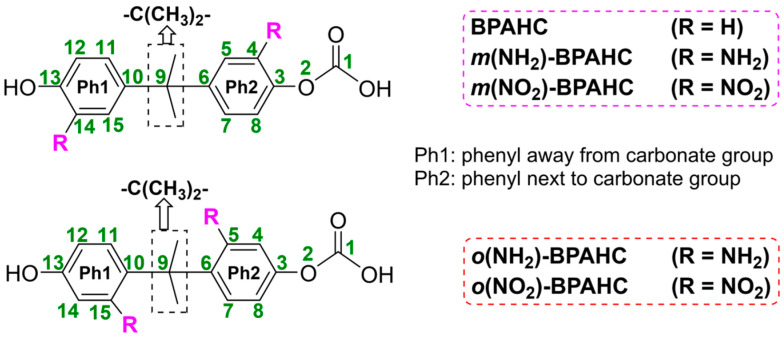
Structures of BPAHC and the substituted BPAHC at -C(CH_3_)_2_-*meta* and -C(CH_3_)_2_-*ortho* positions, signified as *m*(R)-BPAHC and *o*(R)-BPAHC (R=NH_2_, NO_2_) and the corresponding atomic numberings (in green). Partially reproduced from Ref. [17] with permission from the PCCP Owner Societies.

To study the effect of substituent at different positions, Figure 1 shows the structures of the studied PC models with or without substituents. The -C(CH_3_)_2_-*meta* and -C(CH_3_)_2_-*ortho* substituted models are shown at the top and bottom of Figure 1, respectively. In this work, the optimized geometries of the singlet GS and the nth singlet ES are denoted as S_0_ geometry and S_n_ geometry, respectively. The following discussions about the -C(CH_3_)_2_-*meta* substituted models are partially reproduced from Ref. [17]. Notably, as reported in experimental studies [4,9], the O-C bond can be broken at the singlet ES, not the triplet ES. Accordingly, our previous works and the current one only focus on the singlet ES. However, intersystem crossing to the triplet state is still theoretically possible, and it would be worthwhile to include spin–orbit coupling and triplet-state calculations in our next work.

### 2.1. Effect of Electron-Donating -NH_2_ Group on O-C Bond Cleavage

#### 2.1.1. Absorption Spectra in the S_0_ Geometry Under -NH_2_ Effect

**Figure 2:**
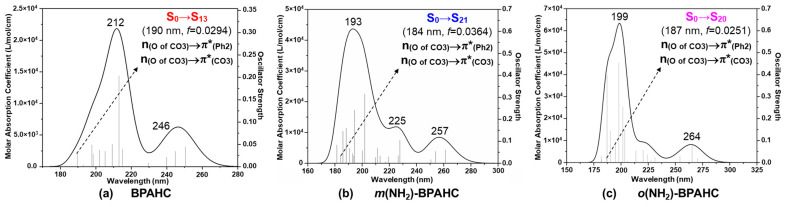
Absorption spectra of BPAHC, *m*(NH_2_)-BPAHC, and *o*(NH_2_)-BPAHC based on their S_0_ geometries. *f* represents the oscillator strength. Partially reproduced from Ref. [17] with permission from the PCCP Owner Societies.

Figure 2 shows the absorption spectra for BPAHC and the -NH_2_ substituted models, *m*(NH_2_)-BPAHC, and *o*(NH_2_)-BPAHC, based on their S_0_ geometries. Table 1 displays the main parameters of the vertical excitation for the focused transitions of BPAHC and the -NH_2_ substituted models. Based on our previous study on BPAHC, the cleavage of the carbonate O-C bond is caused by the S_0_→S_13_ electronic transition, where the electrons are excited from n_(O of CO3)_ to π*_(CO3)_ and π*_(Ph2)_ orbitals. Accordingly, as shown in Figure 2 and Table 1, the S_0_→S_21_ transition for *m*(NH_2_)-BPAHC and S_0_→S_20_ transition for *o*(NH_2_)-BPAHC are examined, as they also mainly involve the n_(O of CO3)_→π*_(Ph2)_ and n_(O of CO3)_→π*_(CO3)_ excitations. The oscillator strengths of the S_0_→S_21_ transition for *m*(NH_2_)-BPAHC and S_0_→S_20_ transition for *o*(NH_2_)-BPAHC are 0.0364 and 0.0251, respectively, which are close to that (0.0294) of the S_0_→S_13_ transition for BPAHC, indicating the comparable contributions to the absorption spectra.

**Figure 3:**
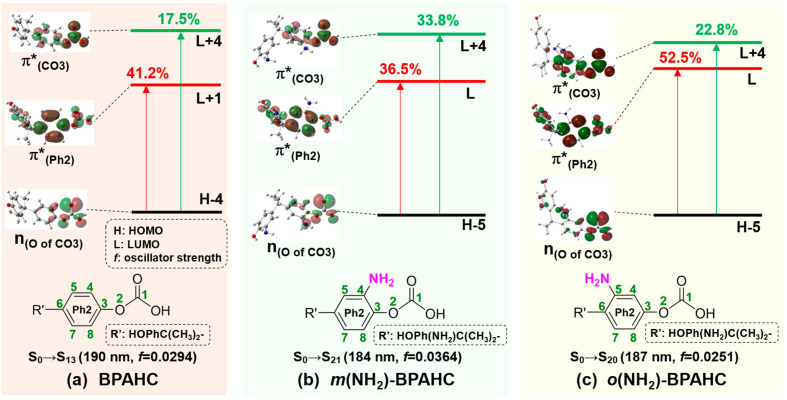
Characteristic MOs (isovalue = 0.03) associated with the vertical excitation of BPAHC, *m*(NH_2_)-BPAHC, and *o*(NH_2_)-BPAHC based on their S_0_ geometries. Percentage values represent the transition contributions. The green numbers represent the atomic numberings. Partially reproduced from Ref. [17] with permission from the PCCP Owner Societies.

To understand the natures of the transitions, Figure 3 exhibits the vertical excitations including the comparisons of the characteristic molecular orbital (MO) for BPAHC, *m*(NH_2_)-BPAHC, and *o*(NH_2_)-BPAHC. In Figure 3, it should be noted that the obtained MO energy levels are dependent on the exchange–correlation functional used, and the variations may take place with different functionals. Compared to the result of BPAHC, there also exist the transitions n_(O of CO3)_→π*_(CO3)_ and n_(O of CO3)_→π*_(Ph2)_ in *m*(NH_2_)-BPAHC and *o*(NH_2_)-BPAHC. Since the MO coefficients of n_(O of CO3)_ and π*_(CO3)_ orbitals mainly concentrate on the carbonate group, their MO shapes are mostly not affected by the -NH_2_ group on the phenyl ring, resulting in the n_(O of CO3)_ and π*_(CO3)_ orbitals in *m*(NH_2_)-BPAHC and *o*(NH_2_)-BPAHC having similar MO shapes to those in BPAHC. However, in *m*(NH_2_)-BPAHC and *o*(NH_2_)-BPAHC, because the MO coefficients of the π*_(Ph2)_ orbital are mainly located on the Ph2 group, its MO shape is greatly influenced by the -NH_2_ group on the phenyl ring and the result of this influence is the generation of a new “nodal plane” on the phenyl ring, leading to the π*_(Ph2)_ orbitals of *m*(NH_2_)-BPAHC and *o*(NH_2_)-BPAHC having different MO shapes from that in BPAHC, as shown in Figure 3. The MO shape of π*_(Ph2)_ orbitals in *m*(NH_2_)-BPAHC and *o*(NH_2_)-BPAHC differs from the π*_(Ph2)_ orbital of BPAHC in that the C4-C5 and C7-C8 in-phase interactions are broken because the C4 (or C5) and C7 (or C8) are exactly located on the new “nodal plane” of π*_(Ph2)_ orbitals in *m*(NH_2_)-BPAHC and *o*(NH_2_)-BPAHC, which corresponds to very small MO coefficients, as shown in Figure 3b,c. Therefore, enhancing the generation of a quinoid-like structure through the C4-C5 and C7-C8 in-phase interactions of π*_(Ph2)_ in BPAHC is not suitable for discussing the O-C bond cleavage in *m*(NH_2_)-BPAHC and *o*(NH_2_)-BPAHC due to the destruction of these in-phase interactions. Nonetheless, the MO coefficients of the π*_(Ph2)_ orbital are mainly focused on the phenyl group that is attached to the carbonate group, so the π*_(Ph2)_ orbital can be used to discuss the substituent effect on O-C bond cleavage although there are no C4-C5 and C7-C8 in-phase interactions of the π*_(Ph2)_ orbital in *m*(NH_2_)-BPAHC and *o*(NH_2_)-BPAHC.

The π*_(Ph2)_ orbital shape in *m*(NH_2_)-BPAHC and *o*(NH_2_)-BPAHC has a significant change due to the generation of a new “nodal plane” of the phenyl ring when there are -NH_2_ groups on phenyl rings as mentioned above, which is different from the π*_(Ph2)_ orbital shape in BPAHC. As shown in Table 1 and Figure 3, the contributions of n_(O of CO3)_→π*_(CO3)_ and n_(O of CO3)_→π*_(Ph2)_ transitions are 17.5% and 41.2% for BPAHC, respectively. Compared to those of BPAHC, for *m*(NH_2_)-BPAHC, the contribution of n_(O of CO3)_→π*_(CO3)_ largely increases to 33.8% while that of n_(O of CO3)_→π*_(Ph2)_ slightly decreases to 36.5%, and both maintain the quite large values, indicating that the O-C bond cleavage is promoted under the effect of the -NH_2_ group at the -C(CH_3_)_2_-*meta* position. For *o*(NH_2_)-BPAHC, the corresponding contributions increase to 22.8% and 52.5%, respectively, implying that the breakage of the O-C bond can also be facilitated by the influence of the -NH_2_ group at the -C(CH_3_)_2_-*ortho* position. Relative to the results of BPAHC and *m*(NH_2_)-BPAHC, it might have a greater promotion effect on the O-C bond cleavage by the -NH_2_ group at the -C(CH_3_)_2_-*ortho* position, since both the contributions of n_(O of CO3)_→π*_(CO3)_ and n_(O of CO3)_→π*_(Ph2)_ transitions are increased in *o*(NH_2_)-BPAHC.

#### 2.1.2. GS and ES Geometries Under -NH_2_ Effect

**Figure 4:**
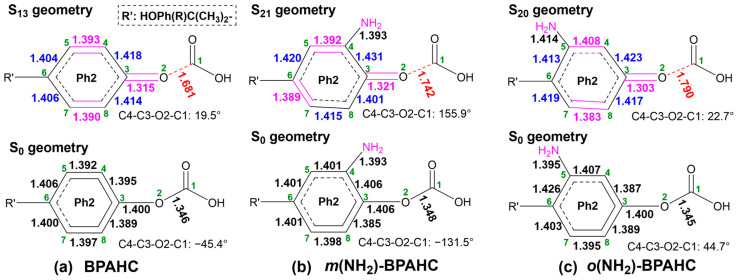
The GS and ES geometries of BPAHC, *m*(NH_2_)-BPAHC, and *o*(NH_2_)-BPAHC along with the primary bond distances (Å). The green numbers represent the atomic numberings. Partially reproduced from Ref. [17] with permission from the PCCP Owner Societies.

Figure 4 presents the comparative analysis of the GS and ES geometries for BPAHC, *m*(NH_2_)-BPAHC, and *o*(NH_2_)-BPAHC to study the effect of the -NH_2_ group at different positions on the O-C bond cleavage.

Following our previous study on BPAHC, the S_13_ geometry is chosen to compare with the S_0_ geometry because the S_0_→S_13_ (BPAHC) transition involves the n_(O of CO3)_→π*_(CO3)_ and n_(O of CO3)_→π*_(Ph2)_ excitations, where the MO coefficients concentrate on the focused carbonate and adjacent Ph2 groups, respectively. Accordingly, the S_21_ geometry for *m*(NH_2_)-BPAHC and the S_20_ geometry for *o*(NH_2_)-BPAHC are chosen to analyze the geometric changes relative to their S_0_ geometry for the same reason.

As shown in Figure 4a, upon excitation, the S_13_ geometry of BPAHC undergoes structural changes compared to the S_0_ geometry, presenting a quinoid-like structure characterized by C4=C5, C7=C8, and C3=O2 double bonds, finally resulting in O2-C1 bond cleavage.

In Figure 4b, when introducing the -NH_2_ group in the -C(CH_3_)_2_-*meta* position, a different quinoid-like structure in the S_21_ geometry of *m*(NH_2_)-BPAHC is formed, with the C4=C5, C6=C7, and C3=O2 double bonds, leading to O2-C1 bond cleavage as in the S_13_ geometry of BPAHC. The possible reason for O2-C1 bond cleavage is that, in *m*(NH_2_)-BPAHC, the formation of a new “nodal plane” along C7-C4-NH_2_ leads to the damage of the C7-C8 in-phase overlap of the π*_(Ph2)_ orbital that exists in BPAHC, as shown in Figure 3. As in the S_0_ geometry, the C4-NH_2_ single bond is retained in the S_21_ geometry, resulting in the alternating single and double bond structure that finally breaks the O2-C1 bond. It indicates that the NH_2_ group at the -C(CH_3_)_2_-*meta* position can cause O2-C1 bond cleavage in the same manner as in the S_13_ geometry of BPAHC.

For *o*(NH_2_)-BPAHC shown in Figure 4c, when the -NH_2_ group is introduced in the -C(CH_3_)_2_-*ortho* position, the S_20_ geometry of *o*(NH_2_)-BPAHC adopts a quinoid-like structure exhibiting C4=C5, C7=C8, and C3=O2 double bonds, finally cleaving the O2-C1 bond. This bond cleavage is attributed to the single and double bond alternations along C6-C3-O2 due to the retention of the single C5-NH_2_ bond in the S_20_ geometry, as in the S_13_ geometry of BPAHC and the S_21_ geometry of *m*(NH_2_)-BPAHC. These results showed that the O2-C1 bond can undergo cleavage affected by the NH_2_ group at the -C(CH_3_)_2_-*ortho* position.

#### 2.1.3. Characteristic MOs Under -NH_2_ Effect

**Figure 5:**
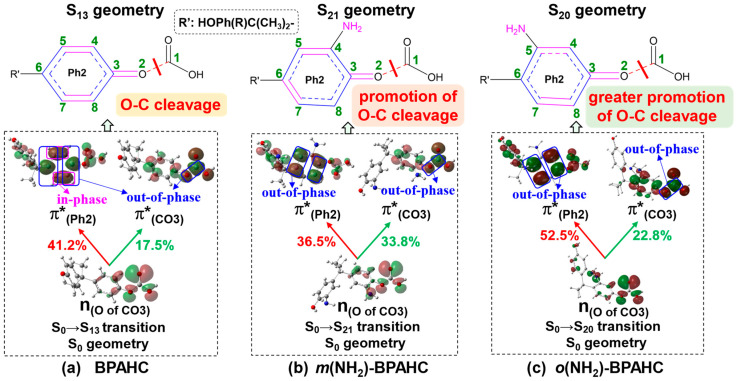
Electronic transitions of BPAHC, *m*(NH_2_)-BPAHC, and *o*(NH_2_)-BPAHC based on their S_0_ geometries, along with the corresponding ES geometries. Percentage values represent the transition contributions. The green numbers represent the atomic numberings. Partially reproduced from Ref. [17] with permission from the PCCP Owner Societies.

To assess how the -NH_2_ group at different positions affects the geometric changes mentioned above, Figure 5 illustrates the comparisons of the characteristic MOs based on their S_0_ geometries for BPAHC, *m*(NH_2_)-BPAHC, and *o*(NH_2_)-BPAHC.

For BPAHC, as shown in Figure 5a, the n_(O of CO3)_→π*_(Ph2)_ excitation enhances the out-of-phase overlaps marked by the blue boxes of π*_(Ph2)_, leading to the elongation of the C3-C4, C3-C8, C5-C6, and C6-C7 bonds. As a result of these C-C elongations, the Ph2 group experiences structural distortion in the S_13_ geometry compared to the S_0_ geometry. The distortion is accompanied by the formation of C4=C5 and C7=C8 bonds through the reinforced C-C in-phase overlaps (marked by the magenta boxes of π*_(Ph2)_), leading to the generation of a quinoid-like structure in the S_13_ geometry. The n_(O of CO3)_→π*_(CO3)_ excitation strengthens the O2-C1 out-of-phase overlap indicated by the blue boxes of π*_(CO3)_, causing the elongation of the O2-C1 bond. The above two excitations result in the cleavage of the O2-C1 bond in the S_13_ geometry.

As in BPAHC, the O2-C1 bond can be cleaved in *m*(NH_2_)-BPAHC as displayed in Figure 4a (top) and Figure 5b. On one hand, the n_(O of CO3)_→π*_(Ph2)_ excitation causes the extension of C3-C8 and C5-C6 bonds due to the enhancement of C-C out-of-phase overlaps indicated by the blue boxes of π*_(Ph2)_. The C-C bond extensions lead to the distortion of the Ph2 group in the S_21_ geometry compared to the S_0_ geometry, in which the C4=C5 and C6=C7 double bonds within Ph2 group are produced. Subsequently, a quinoid-like structure having C4=C5, C6=C7, and C3=O2 double bonds is generated. On the other hand, the n_(O of CO3)_→π*_(CO3)_ excitation causes O2-C1 bond elongation since the out-of-phase overlap indicated by the blue box of π*_(CO3)_ is strengthened. The above two excitations jointly cleave the O2-O1 bond in the S_21_ geometry. Moreover, the significant increase in the n_(O of CO3)_→π*_(CO3)_ transition contribution (33.8%), compared to 17.5% for BPAHC, indicates a greater tendency to lengthen the O2-C1 bond. It means that the promotion of the O2-C1 bond cleavage is affected by the -NH_2_ group at the -C(CH_3_)_2_-*meta* position.

O2-C1 bond cleavage also takes place in *o*(NH_2_)-BPAHC, caused by the n_(O of CO3)_→π*_(Ph2)_ and n_(O of CO3)_→π*_(CO3)_ excitations, as shown in Figure 5c. When the electron is excited to the π*_(Ph2)_ orbital, the C3-C4 and C6-C7 bonds are elongated, since the C-C out-of-phase overlaps of π*_(Ph2)_ (marked by the blue boxes of π*_(Ph2)_) are enhanced. Due to these C-C bond extensions, the Ph2 group becomes distorted in the S_20_ geometry compared to the S_0_ geometry, leading to the C4=C5 and C7=C8 double bonds. Consequently, the S_20_ geometry presents a tendency toward a quinoid-like structure, featuring C4=C5, C7=C8, and C3=O2 double bonds. When the electron is excited to π*_(CO3)_, the O2-C1 bond is elongated as the out-of-phase overlaps of π*_(CO3)_ (indicated by the blue boxes of π*_(CO3)_) are strengthened. Additionally, the contributions of n_(O of CO3)_→π*_(CO3)_ and n_(O of CO3)_→π*_(Ph2)_ transitions increase to 22.8% and 52.5%, relative to those of 17.5% and 41.2% in BPAHC, respectively, indicating a larger driving force to break the O2-C1 bond. It means that the O2-C1 bond cleavage is facilitated by introducing the -NH_2_ group in the -C(CH_3_)_2_-*ortho* position. In comparison to the results of BPAHC, one transition contribution increases for *m*(NH_2_)-BPAHC while both the contributions of the two focused transitions increase for *o*(NH_2_)-BPAHC. It implies that the introduction of the -NH_2_ group at the -C(CH_3_)_2_-*ortho* position is more significant in facilitating the O2-C1 bond cleavage than at the -C(CH_3_)_2_-*meta* position.

#### 2.1.4. Potential Energy Surfaces (PESs) Under -NH_2_ Effect

**Figure 6:**
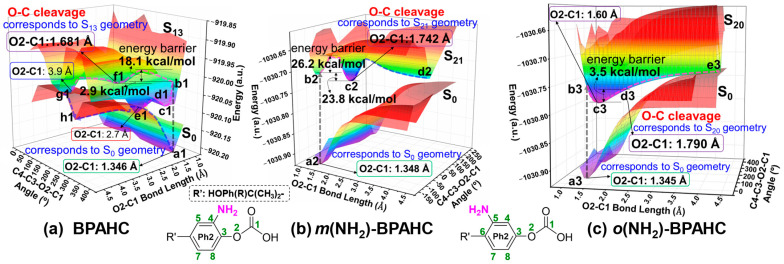
PESs for the GS and ES of BPAHC, *m*(NH_2_)-BPAHC, and *o*(NH_2_)-BPAHC, with respect to the O2-C1 bond distance and C4-C3-O2-C1 dihedral angle. The green numbers represent the atomic numberings. Partially reproduced from Ref. [17] with permission from the PCCP Owner Societies.

The PESs of the GS and ES are obtained using density functional theory (DFT) and time-dependent DFT (TDDFT) methods to evaluate the effect of -NH_2_ on the O-C bond cleavage. As described above, there is a possible dissociative behavior of the ES geometries along the O2-C1 bond which might lead to the breakage of the carbonate plane, so the O2-C1 bond and the C4-C3-O2-C1 dihedral angle (between carbonate and Ph2 planes) are chosen as the functions to perform the PES scans of the GS and ES for *m*(NH_2_)-BPAHC and *o*(NH_2_)-BPAHC compared to those of BPAHC, as shown in Figure 6.

Referring to our previous study on the PESs of BPAHC, points a1 and f1 correspond to the S_0_ and S_13_ states, respectively, and the corresponding O2-C1 bond distances are 1.346 Å and 1.681 Å, as shown in Figure 6a. The predicted energy barrier is about 18.1 kcal/mol for b1→f1 on the S_13_ PES when the breakage of the O-C bond occurs, as shown in Figure 6a. In addition, the radicals Ph=O• and •COOH generated from the O-C cleavage can further interact, leading to either recombination or separation on the S_0_ PES via the intersections (points e1 and g1) with the S_13_ PES.

As for the PESs of *m*(NH_2_)-BPAHC, the O2-C1 bond distances at points a2 and c2 are 1.348 Å and 1.742 Å (see Figure 6b), respectively, which correspond to the S_0_ and S_21_ states. The PES analysis of the S_21_ state in *m*(NH_2_)-BPAHC indicates that the O-C bond cleavage proceeds through an estimated energy barrier of 26.2 kcal/mol. Although this value is somewhat higher than in BPAHC, the impact on the O-C bond cleavage remains unclear due to the limitation of the coarse grid calculations. The energy of point c2 is lower by 23.8 kcal/mol relative to that of point b2. It indicates that it is easier to reach the local minimum at point c2, where the O2-C1 bond is broken. In addition, the absence of the intersection between the S_0_ and S_21_ PESs suggest that the O2∙··C1 bond is more susceptible to breaking due to its hindered recombination. These results reveal that the NH_2_ groups at the -C(CH_3_)_2_-*meta* positions can facilitate the carbonate O-C bond cleavage compared to BPAHC.

On the PESs of *o*(NH_2_)-BPAHC, the O2-C1 bond distances of the S_0_ (point a3) and S_20_ (point d3) states are 1.4 Å and 1.79 Å, respectively, as depicted in Figure 6c. Relative to the predicted energy barriers for the O-C bond cleavage in BPAHC and *m*(NH_2_)-BPAHC, it has a lower energy barrier of about 3.5 kcal/mol for c3→d3 on the S_20_ PES of *o*(NH_2_)-BPAHC, indicating that the O2-C1 bond is prone to be broken. It should be mentioned here that point d3 of the S_20_ PES of *o*(NH_2_)-BPAHC is not located at a minimum, while the point c3 with the O2-C1 bond distance of 1.6 Å is located at a minimum, which is different from the PESs of BPAHC and *m*(NH_2_)-BPAHC. This may be due to the rough PES scan. However, both the relative energies and the O2-C1 bond distance are close to each other at points c3 and d3, so here the point d3 that corresponds to the S_20_ state of *o*(NH_2_)-BPAHC is used for discussion. Moreover, for the same reason as in *m*(NH_2_)-BPAHC, nonexistence of an intersection between these two PESs leads to the O2-C1 bond being damaged more easily. Therefore, relative to BPAHC, the NH_2_ groups at the -C(CH_3_)_2_-*ortho* positions can also promote O-C bond damage.

### 2.2. Effect of Electron-Withdrawing -NO_2_ Group on O-C Bond Cleavage

#### 2.2.1. Absorption Spectra in the S_0_ Geometry Under -NO_2_ Effect

**Figure 7:**
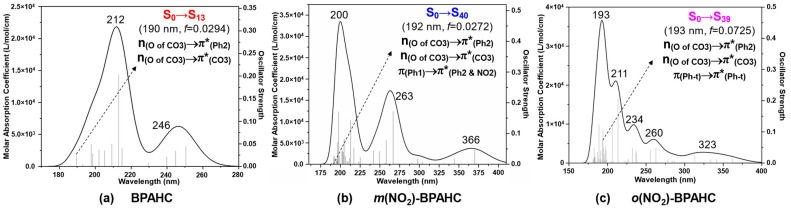
Absorption spectra of BPAHC, *m*(NO_2_)-BPAHC, and *o*(NO_2_)-BPAHC based on their S_0_ geometries. *f* represents the oscillator strength. Partially reproduced from Ref. [17] with permission from the PCCP Owner Societies.

To assess the effect of the -NO_2_ group at the different positions on the O-C bond cleavage, Figure 7 shows the predicted absorption spectra of BPAHC, *m*(NO_2_)-BPAHC, and *o*(NO_2_)-BPAHC. Table 2 displays the primary vertical parameters for the concerned transitions of BPAHC, *m*(NO_2_)-BPAHC, and *o*(NO_2_)-BPAHC. As indicated by our earlier study on BPAHC and *m*(NO_2_)-BPAHC, under the effect of the -NO_2_ group at the -C(CH_3_)_2_-*meta* position, the excitations to π*_(CO3)_, π*_(Ph2)_, and π*_(Ph2 & NO2)_ can cause the formation of the quinoid-like structure, finally suppressing the O-C bond cleavage. The transitions referring to Ph2 and adjacent carbonate groups in BPAHC, *m*(NO_2_)-BPAHC, and *o*(NO_2_)-BPAHC are chosen to examine the effect of -NO_2_ on the O-C bond cleavage, which is the same as in the case of the effect of -NH_2_.

As shown in Figure 7 and Table 2, as for the effect of -NO_2_ at -C(CH_3_)_2_-*meta* and -C(CH_3_)_2_-*ortho* positions, corresponding to the S_0_→S_13_ transition of BPAHC, there are the S_0_→S_40_ transition at 192 nm for *m*(NO_2_)-BPAHC and the S_0_→S_39_ transition at 193 nm for *o*(NO_2_)-BPAHC, because both transitions include the n_(O of CO3)_→π*_(Ph2)_ and n_(O of CO3)_→π*_(CO3)_ excitations as in BPAHC. The S_0_→S_40_ transition of *m*(NO_2_)-BPAHC is mainly from the n_(O of CO3)_→π*_(Ph2)_ with a contribution of 42.6% and the π_(Ph1)_→π*_(Ph2 & NO2)_ with a contribution of 24.3%. The S_0_→S_39_ transition of *o*(NO_2_)-BPAHC is attributed to the promotion of π_(Ph-t)_→π*_(Ph-t)_ with a 20.7% contribution and n_(O of CO3)_→π*_(Ph2)_ with a 10.5% contribution.

For the same reason as mentioned in Section 2.1, the C4-C5 and C7-C8 in-phase interactions of the π*_(Ph2)_ orbital in *m*(NO_2_)-BPAHC and *o*(NO_2_)-BPAHC are broken because of the generation of a new “nodal plane” affected by the -NO_2_ group, and the MO coefficients of the π*_(Ph2)_ orbitals in *m*(NO_2_)-BPAHC and *o*(NO_2_)-BPAHC mainly concentrate on the Ph2 group, thus the π*_(Ph2)_ orbitals in *m*(NH_2_)-BPAHC and *o*(NH_2_)-BPAHC are used to evaluate the effect of the -NO_2_ group on O-C bond cleavage as in *m*(NH_2_)-BPAHC and *o*(NH_2_)-BPAHC.

**Figure 8:**
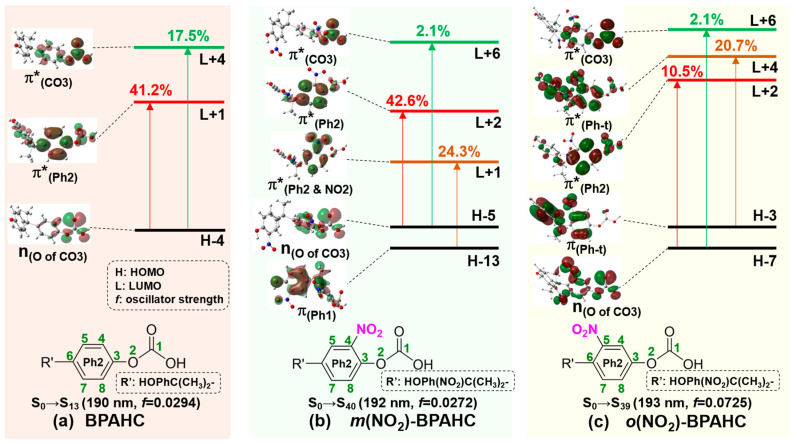
Characteristic MOs (isovalue = 0.03) for BPAHC, *m*(NO_2_)-BPAHC, and *o*(NO_2_)-BPAHC based on their S_0_ geometries. The green numbers represent the atomic numberings. Percentage values represent the transition contribution. Partially reproduced from Ref. [17] with permission from the PCCP Owner Societies.

To elucidate the nature of the transition, Figure 8 shows the vertical excitations and the comparison of the characteristic MOs for BPAHC, *m*(NO_2_)-BPAHC, and *o*(NO_2_)-BPAHC. Upon excitation, in BPAHC shown in Figure 8a, the contributions of n_(O of CO3)_→π*_(CO3)_ and n_(O of CO3)_→π*_(Ph2)_ transitions are 17.5% and 41.2%, respectively. Accordingly, in *m*(NO_2_)-BPAHC as shown in Figure 8b, the n_(O of CO3)_→π*_(CO3)_ contribution sharply drops to 2.1% while the n_(O of CO3)_→π*_(Ph2)_ contribution maintains a large value of 42.6%. Additionally, another π_(Ph1)_→π*_(Ph2 & NO2)_ transition with a contribution of 24.3% is included. However, in *o*(NO_2_)-BPAHC as shown in Figure 8c, both the n_(O of CO3)_→π*_(CO3)_ and n_(O of CO3)_→π*_(Ph2)_ transition contributions largely decrease to 2.1% and 10.5%, respectively, although a new π_(Ph-t)_→π*_(Ph-t)_ transition appears with a contribution of 20.7%. Compared to the results of BPAHC, the presence of the -NO_2_ group, regardless of being at the -C(CH_3_)_2_-*meta* or -C(CH_3_)_2_-*ortho* position, can suppress the O-C bond cleavage. In addition, the -NO_2_ group at the -C(CH_3_)_2_-*ortho* position can more significantly suppress the O-C bond cleavage than that at the -C(CH_3_)_2_-*meta* position, because both the contributions of n_(O of CO3)_→π*_(CO3)_ and n_(O of CO3)_→π*_(Ph2)_ excitations are decreased relative to those of BPAHC.

#### 2.2.2. GS and ES Geometries Under -NO_2_ Effect

**Figure 9:**
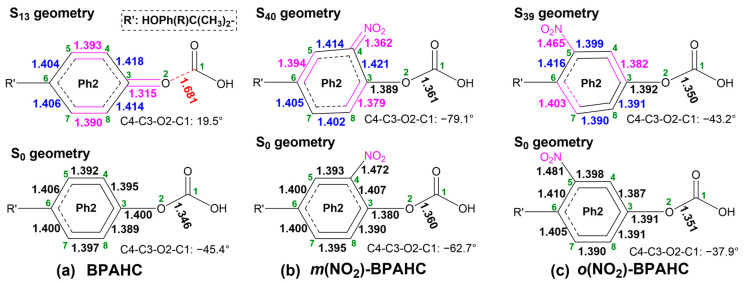
GS and ES geometries of BPAHC, *m*(NO_2_)-BPAHC, and *o*(NO_2_)-BPAHC along with the primary bond distances (Å). The green numbers represent the atomic numberings. Partially reproduced from Ref. [17] with permission from the PCCP Owner Societies.

Figure 9 presents the structural comparison of GS and ES geometries for BPAHC, *m*(NO_2_)-BPAHC, and *o*(NO_2_)-BPAHC in order to investigate the influence of the -NO_2_ groups at different positions on the breakage of the O-C bond.

As shown in Figure 9a, relative to the S_0_ geometry of BPAHC, the S_13_ geometry becomes a quinoid-like structure along with C4=C5, C7=C8, and O2=C3 double bonds by the excitation, breaking the O2-C1 bond.

Different from the results of BPAHC, as shown in Figure 9b, with the introduction of -NO_2_ groups in -C(CH_3_)_2_-*meta* positions, upon the electronic excitation, the S_40_ geometry tends to be a new quinoid-like structure with C5=C6, C3=C8, and C4=NO_2_ double bonds, keeping the O2-C3 single bond, preventing the O2-C1 bond cleavage. This may be attributed to the damage of the C4-C5 and C7-C8 in-phase overlaps of π*_(Ph2)_ in *m*(NO_2_)-BPAHC, which are present in BPAHC, as shown in Figure 8. The C=NO_2_ bond formation caused by the in-phase overlap of π*_(Ph2 & NO2)_ (see Figure 8b) leads to single and double bond alternation that hinders the breakage of the O2-C1 bond. Hence, the O2-C1 single bond is not broken under the effect of the -NO_2_ groups at -C(CH_3_)_2_-*meta* positions.

For *o*(NO_2_)-BPAHC, with the incorporation of -NO_2_ groups in -C(CH_3_)_2_-*ortho* positions, the S_39_ geometry takes on another quinoid-like structure with C3=C4 and C6=C7 double bonds along C8-C5-NO_2_, maintaining the O2-C3 single bond and avoiding the O2-C1 bond cleavage, as shown in Figure 9c. The formations of C3=C4 and C6=C7 bonds are caused by the C-C in-phase overlaps of the π*_(Ph-t)_ orbital (see Figure 8c). Different from *m*(NO_2_)-BPAHC, in *o*(NO_2_)-BPAHC, the C5-NO_2_ bond of the S_39_ geometry (1.465 Å) has a reduction relative to that in its S_0_ geometry (1.481 Å), but both maintain the single bond nature. Nevertheless, the π_(Ph-t)_→π*_(Ph-t)_ excitation plays a major role due to a higher transition contribution than the other two excitations (see Figure 8c), enabling the single and double bond alternations along C8-C5-NO_2_, preventing the O2-C1 bond from breaking. The results show that the O2-C1 single bond is also kept when introducing the -NO_2_ groups in the -C(CH_3_)_2_-*ortho* positions.

#### 2.2.3. Characteristic MOs Under -NO_2_ Effect

**Figure 10:**
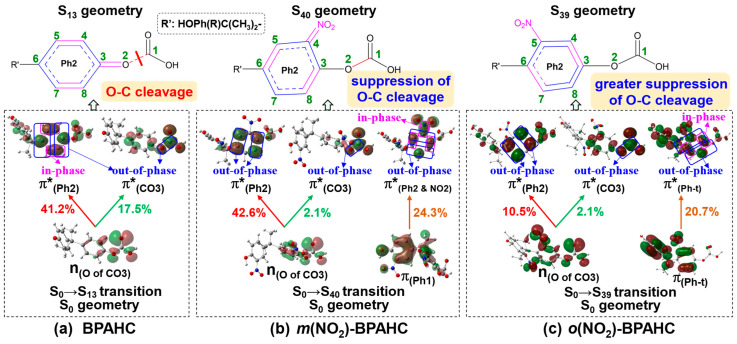
Electronic transitions of BPAHC, *m*(NO_2_)-BPAHC, and *o*(NO_2_)-BPAHC based on their S_0_ geometries along with the corresponding ES geometries. Percentage values represent the transition contributions. The green numbers represent the atomic numberings. Partially reproduced from Ref. [17] with permission from the PCCP Owner Societies.

Figure 10 illustrates the MO comparisons based on their S_0_ geometries for BPAHC, *m*(NO_2_)-BPAHC, and *o*(NO_2_)-BPAHC to explain the above geometric alternations affected by the -NO_2_ groups at different positions.

In BPAHC, as shown in Figure 10a, the n_(O of CO3)_→π*_(Ph2)_ and n_(O of CO3)_→π*_(CO3)_ excitations cause the formation of a quinoid-like structure, finally breaking the O2-C1 bond in the S_13_ geometry. The details are shown in Section 2.1.

In contrast, for *m*(NO_2_)-BPAHC as depicted in Figure 10b, the O2-C1 maintains its single bond nature in the S_40_ geometry, just as it is in the S_0_ geometry. This can be explained by the following reasons. First, the strengthening of the C4-NO_2_ in-phase overlap, caused by the newly appeared n_(O of CO3)_→π*_(Ph2 & NO2)_ excitation, leads to the significant shortening of the C4-NO_2_ bond, along with the contractions of C5-C6 and C3-C8 bonds. Second, the reinforcement of the C-C out-of-phase overlaps, caused by the n_(O of CO3)_→π*_(Ph2)_ excitation, results in the elongations of C5-C6 and C3-C8 bonds. The n_(O of CO3)_→π*_(Ph2 & NO2)_ excitation has a transition contribution of 24.3% which is lower than that (42.6%) of the n_(O of CO3)_→π*_(Ph2)_ excitation, indicating that the first one shows a smaller influence than the second one. However, the effect of the first one is reflected in the S_40_ geometry, whereas that of the second one is absent. Considering the above two reasons, the observed C3=C8 and C5=C6 bonds indicate that the bond alternation is primarily driven by the geometric impact of the C=NO_2_ bond which is caused by the enhanced C4-NO_2_ in-phase overlap, rather than the reinforced C-C out-of-phase overlaps. Finally, both the excitations lead to the formation of the quinoid-like structure with C5=C6, C3=C8, and C4=NO_2_ double bonds, keeping the O2-C3 single bond nature and avoiding the O2-C1 bond being broken. In addition, compared to the results of BPAHC, the contribution of the n_(O of CO3)_→π*_(Ph2)_ transition maintains a large value of 42.6%, whereas that of the n_(O of CO3)_→π*_(CO3)_ transition sharply decreases to 2.1%, indicating that the O1-C2 bond cleavage is suppressed under the effect of -C(CH_3_)_2_-*meta* substituted -NO_2_ groups. These results indicate that the -NO_2_ groups at -C(CH_3_)_2_-*meta* positions can lead to the suppression of the O2-C1 bond cleavage.

As in *m*(NO_2_)-BPAHC, the O2-C1 single bond is also maintained in the S_39_ geometry of *o*(NO_2_)-BPAHC relative to the S_0_ geometry, as shown in Figure 10c. It can be clarified by the following explanation. The excitation to π*_(Ph2)_ leads to the elongation of C6-C7 and C3-C4 bonds and the corresponding C-C out-of-phase overlaps are enhanced. On the contrary, the excitation to π*_(Ph-t)_ leads to the reduction of C6-C7 and C3-C4 bonds due to the strengthened C-C in-phase overlaps, while the elongation of C4-C5, C5-C6, C7-C8, and C3-C8 bonds owes to the enhanced C-C out-of-phase overlaps. Notably, upon the excitation to π*_(Ph2)_ and π*_(Ph-t)_, there are the contrary effects on the C6-C7 and C3-C4 bonds. However, the n_(O of CO3)_→π*_(Ph-t)_ excitation has a higher contribution of 20.7% than that (10.5%) of the n_(O of CO3)_→π*_(Ph2)_ excitation, indicating that the n_(O of CO3)_→π*_(Ph-t)_ excitation shows a greater impact. It means that the C6-C7 and C3-C4 bonds will be shortened by these excitations. Due to the geometric limitations caused by the decreased C6-C7 and C3-C4 bonds, the C4-NO_2_ bond is shortened in the S_39_ geometry, preventing the O1-C2 bond from breaking under the effect of -C(CH_3_)_2_-*ortho* substituted -NO_2_ groups. Relative to the transition contributions of BPAHC, the contributions of the n_(O of CO3)_→π*_(Ph2)_ and n_(O of CO3)_→π*_(CO3)_ excitations significantly decrease to 10.5% and 2.1%, respectively, indicating that the -NO_2_ groups at -C(CH_3_)_2_-*meta* positions can suppress the O1-C2 bond cleavage. Furthermore, the contribution of the n_(O of CO3)_→π*_(Ph2)_ excitation (10.5%) of *o*(NO_2_)-BPAHC decreases more while that of *m*(NO_2_)-BPAHC maintains a large value of 42.6%, implying that the O1-C2 bond cleavage can be suppressed more significantly when introducing the -NO_2_ groups in the -C(CH_3_)_2_-*ortho* positions.

#### 2.2.4. PESs Under -NO_2_ Effect

**Figure 11:**
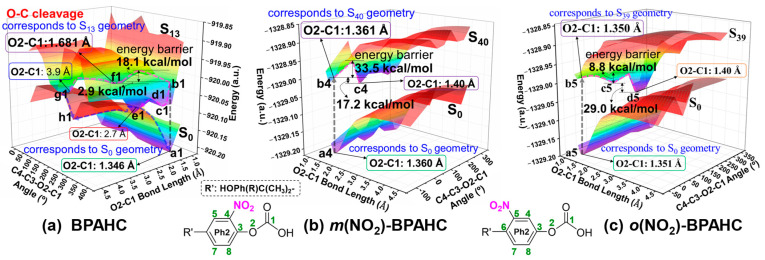
PESs for the GS and ES of BPAHC, *m*(NO_2_)-BPAHC, and *o*(NO_2_)-BPAHC, with respect to the O2-C1 bond distance and C4-C3-O2-C1 dihedral angle. The green numbers represent the atomic numberings. Partially reproduced from Ref. [17] with permission from the PCCP Owner Societies.

For the same reason as in Section 2.1, Figure 11 shows the PESs of the GS and ES for *m*(NO_2_)-BPAHC and *o*(NO_2_)-BPAHC to further analyze the substituent effect on the O-C bond cleavage.

Figure 11a displays our earlier study of the PESs of BPAHC, which is also mentioned in Section 2.1. As shown in Figure 11a, there is an energy barrier of around 18.1 kcal/mol (from points b1 to f1 on the S_13_ PES) that can be readily surpassed to break the O-C bond. In addition, the intersections (points e1 and g1) between the S_0_ and S_13_ PESs indicate that the radicals Ph=O• and •COOH could recombine or separate on the S_0_ PES starting from points e1 and g1.

On the PESs of *m*(NO_2_)-BPAHC, as shown in Figure 11b, point a4 on the S_0_ PES and point b4 on the S_40_ PES match the S_0_ geometry and the S_40_ geometry, respectively. The O2-C1 bond distance in the S_40_ geometry is 1.361 Å which is similar to that (1.360 Å) in the S_0_ geometry, indicating that the O2-C1 bond is not broken in *m*(NO_2_)-BPAHC compared to BPAHC. This can be clarified from the following two aspects. On one hand, from points b4 to c4 on the S_40_ PES, a very higher energy barrier of about 33.5 kcal/mol is required relative to BPAHC. It indicates that reaching the local minimum of point c4 from point b4 is challenging, even though its energy is lower by 17.2 kcal/mol than that of point b4. On the other hand, in contrast to the case of BPAHC, the S_0_ and S_40_ PESs do not intersect, suggesting that the recombination of the broken O2···C1 bond is impossible. Finally, the O2-C1 bond maintains its single bond nature in the S_40_ geometry as in the S_0_ geometry. These results show that the O-C bond cleavage can be suppressed under the influence of -NO_2_ groups at -C(CH_3_)_2_-*meta* positions.

Likewise, for the *o*(NO_2_)-BPAHC, the O2-C1 single bond is maintained in the ES (S_39_) geometry just as in its corresponding GS (S_0_) geometry (see Figure 9c), which is explained as follows. On the PESs of the S_0_ and S_39_ states as displayed in Figure 11c, points a5 and b5 correspond to the S_0_ and S_39_ geometries, respectively, where the O2-C1 bond distances are about 1.35 Å. Relative to the local minimum of the S_39_ state, there are the other two local minima at points c5 and d5 on the S_39_ PES, which are easily accessible due to the lack of a barrier for b5→c5 and the low energy barrier of about 8.8 kcal/mol for c5→d5. This is different from the above situation of *m*(NO_2_)-BPAHC. However, the S_39_ state at point b5, rather than the local minima at points c5 and d5, is chosen for discussion, because it may be also a local minimum due the flat S_39_ PES for b5→c5 under the current rough calculations. As observed in *m*(NO_2_)-BPAHC, there is also no intersection between the S_0_ and S_39_ PESs, indicating that it is impossible to recombine the broken O2···C1 bond. Hence, the O-C bond cleavage is also suppressed under the impact of -NO_2_ groups at -C(CH_3_)_2_-*ortho* positions.

## 3. Computational Details

DFT and TDDFT [18] calculations were carried out on the substituted BPAHC models *o*(NH_2_)-BPAHC and *o*(NO_2_)-BPAHC (see Figure 1). The geometry optimizations were conducted on the GSs and ESs without any restrictions using the B3LYP [19,20,21]/6-31G(d) and TD-B3LYP/6-31G(d) methods, respectively, which could provide reliable results in characterizing this organic system [22,23]. All calculations were conducted in the gas phase because PC is usually studied in the solid state in order to concentrate on the intrinsic electronic structure of the studied PC models. The absorption spectra were depicted adopting Multiwfn 3.8 [24] and Origin 9.1 [25] software, which were convoluted with a Gaussian function using a full width at half maximum of 0.38 eV. The MO representations were generated with GaussView 6.1.1 [26]. All calculations in this work were conducted with the Gaussian 16 program package [27].

## 4. Conclusions

Collectively, the effect of substituents of phenyl rings on PC carbonate O-C bond cleavage was investigated based on DFT and TDDFT methods using simplified PC models with -NH_2_ or -NO_2_ groups. The n_(O of CO3)_→π*_(Ph2)_ and n_(O of CO3)_→π*_(CO3)_ transitions contribute to the quinoid-like structure formation and the carbonate O-C bond extension, respectively, finally causing the breakage of the carbonate O-C bond.

Compared to the results of BPAHC, in *m*(NH_2_)-BPAHC, the -C(CH_3_)_2_-*meta* substituted -NH_2_ groups can promote carbonate O-C bond scission because of the sustained high n_(O of CO3)_→π*_(Ph2)_ transition contribution and the intensified n_(O of CO3)_→π*_(CO3)_ transition contribution. In *o*(NH_2_)-BPAHC, the -C(CH_3_)_2_-*ortho* substituted -NH_2_ groups facilitate the O-C bond cleavage more significantly due to the increased contributions of both the above transitions. Moreover, the difficulties in remaking the broken O∙∙∙C bond, caused by the absence of intersections between the PESs of the GS and ES, further confirm these promotions.

Contrarily, in *m*(NO_2_)-BPAHC, the -C(CH_3_)_2_-*meta* substituted -NO_2_ groups inhibit the O-C bond breakage because only the contribution of the n_(O of CO3)_→π*_(CO3)_ transition has a very large decrease relative to that of BPAHC. In *o*(NO_2_)-BPAHC, the -C(CH_3_)_2_-*ortho* substituted -NO_2_ groups suppress the O-C bond scission more significantly, since both of the above transition contributions have remarkable reductions. These suppressions are reflected in the maintenance of the O-C single bonds at the potential minima on the PESs of ESs.

In a word, the promotion and suppression of carbonate O-C bond scission are enhanced when introducing the substituents in the -C(CH_3_)_2_-*ortho* positions. This means, to facilitate PC photodegradation, it would be better to place the electron-donating groups at the -C(CH_3_)_2_-*ortho* positions; conversely, to promote PC photostability, it would be better to place the electron-withdrawing groups at the same positions.

These results offer a thorough understanding and a practical approach for the design of degradable PC materials or the development of UV-resistant PC materials, and future experimental validation would be crucial to confirm their broader applications. Moving forward, we expect to explore the effects like chain length, interchain interaction, and stabilizers in large systems using frontier molecular orbitalets (FMOLs) [28], which may be a useful tool to analyze the local excited frontier molecular orbitals obtained in our previous study [29].

## Figures and Tables

**Table 1:** Main vertical parameters for BPAHC, *m*(NH_2_)-BPAHC, and *o*(NH_2_)-BPAHC based on their S_0_ geometries, and only the contribution greater than 10% is shown. Partially reproduced from Supplementary Information of Ref. [17] with permission from the PCCP Owner Societies.

	Electronic Transition	Energy (eV)	λ (nm)	*f* *^a^*	Contribution (%)	Transition *^b^*	Assignment
BPAHC	S_0_→S_13_	6.54	190	0.0294	41.2	H-4→L+1	n_(O of CO3)_→π*_(Ph2)_
					17.5	H-4→L+4	n_(O of CO3)_→π*_(CO3)_
					15.1	H-3→L+1	π_(Ph2)_→π*_(Ph2)_
*m*(NH_2_)-BPAHC	S_0_→S_21_	6.73	184	0.0364	36.5	H-5→L	n_(O of CO3)_→π*_(Ph2)_
					33.8	H-5→L+4	n_(O of CO3)_→π*_(CO3)_
*o*(NH_2_)-BPAHC	S_0_→S_20_	6.65	187	0.0251	52.5	H-5→L	n_(O of CO3)_→π*_(Ph2)_
					22.8	H-5→L+4	n_(O of CO3)_→π*_(CO3)_

*^a^* Oscillator strength. *^b^* H and L represent the highest occupied molecular orbital (HOMO) and the lowest unoccupied molecular orbital (LUMO), respectively.

**Table 2:** Main vertical parameters for BPAHC, *m*(NO_2_)-BPAHC, and *o*(NO_2_)-BPAHC based on their S_0_ geometries, and only contributions greater than 10% are shown here except for *m*(NO_2_)-BPAHC and *o*(NO_2_)-BPAHC. Partially reproduced from Supplementary Information of Ref. [17] with permission from the PCCP Owner Societies.

	Electronic Transition	Energy (eV)	λ (nm)	*f* ^*a*^	Contribution (%)	Transition *^b^*	Assignment
BPAHC	S_0_→S_13_	6.54	190	0.0294	41.2	H-4→L+1	n_(O of CO3)_→π*_(Ph2)_
					17.5	H-4→L+4	n_(O of CO3)_→π*_(CO3)_
					15.1	H-3→L+1	π_(Ph2)_→π*_(Ph2)_
*m*(NO_2_)-BPAHC	S_0_→S_40_	6.46	192	0.0272	42.6	H-5→L+2	n_(O of CO3)_→π*_(Ph2)_
					2.1	H-5→L+6	n_(O of CO3)_→π*_(CO3)_
					24.3	H-13→L+1	π_(Ph1)_→π*_(Ph2 & NO2)_
*o*(NO_2_)-BPAHC	S_0_→S_39_	6.42	193	0.0725	10.5	H-7→L+2	n_(O of CO3)_→π*_(Ph2)_
					2.1	H-7→L+6	n_(O of CO3)_→π*_(CO3)_
					20.7	H-3→L+4	π_(Ph-t)_→π*_(Ph-t)_

*^a^* Oscillator strength. *^b^* H and L represent HOMO and LUMO, respectively.

## Data Availability

The data presented in this work are available in the article.
